# Supplementary material for: In Situ Synthesis of CsPbX3/Polyacrylonitrile Nanofibers with Water-Stability and Color-Tunability for Anti-Counterfeiting and LEDs
Source: Polymers (Basel). 2024 Jun 1;16(11):1568. doi: 10.3390/polym16111568 (PMC11174623; doi:10.3390/polym16111568)
Supplement: Supplementary file 1 [file polymers-16-01568-s001.zip › polymers-3028831-supplementary.pdf]

Supplementary Materials

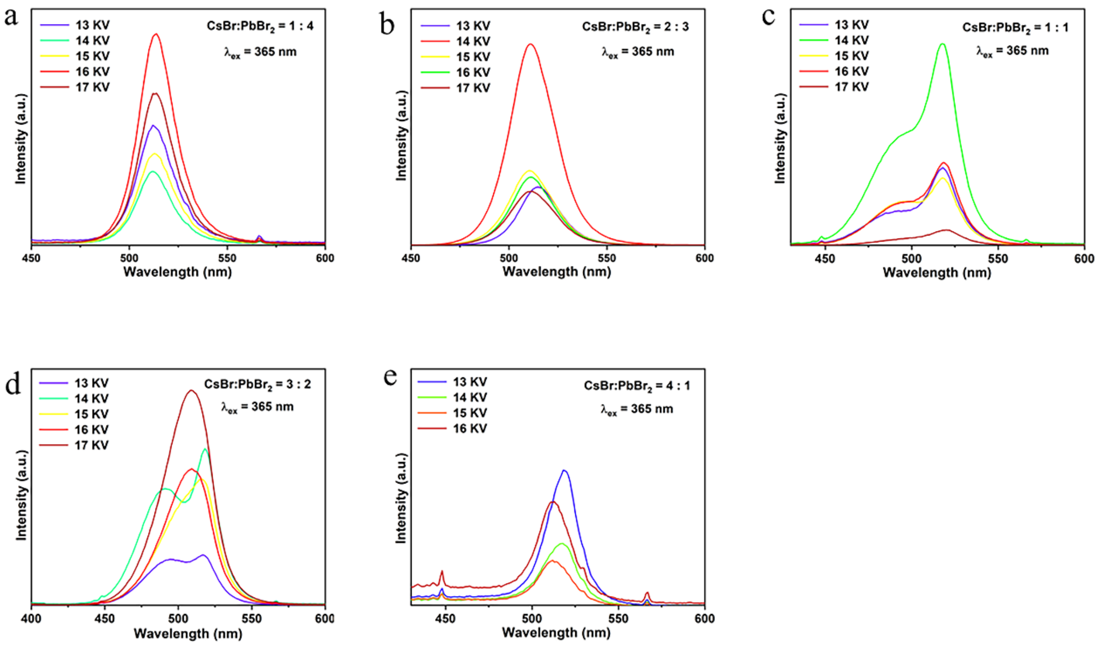

Figure S1. (a-e) PL spectra of $CsPbBr_3$/PAN composite nanofibers under different voltages and different ratios of CsBr and $PbBr_2$.

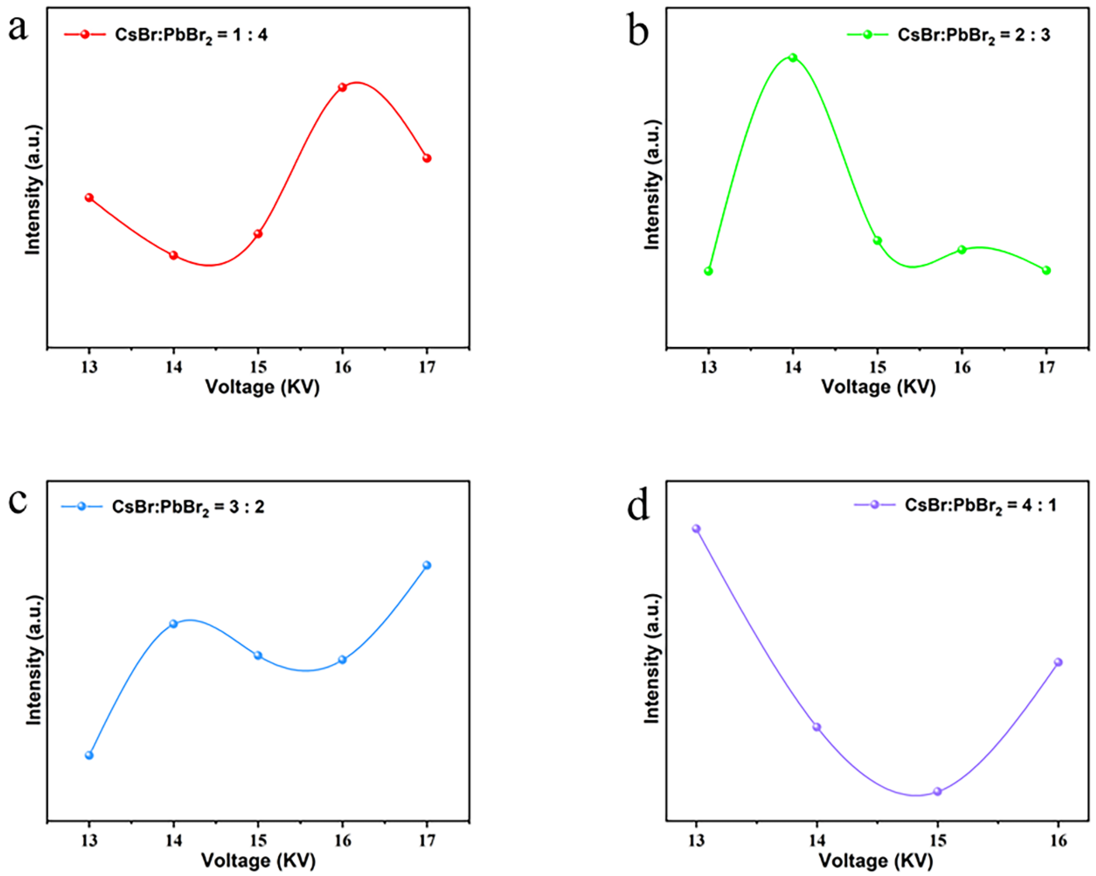

Figure S2. (a-d) PL Intensity trend change chart of $CsPbBr_3$/PAN composite nanofibers under different voltages and different ratios of CsBr and $PbBr_2$.

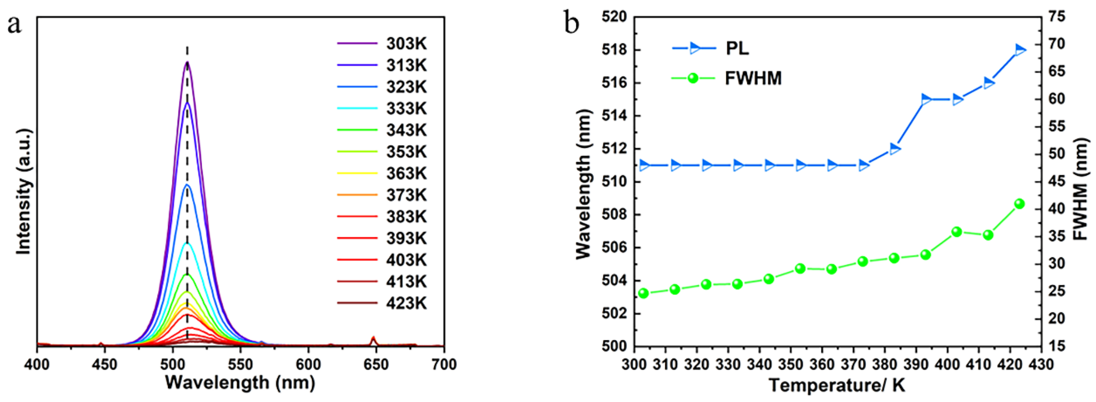

Figure S3. (a) Temperature-varying PL spectra of $CsPbBr_3$/PAN composite film. (b) PL peak positons and FWHM.

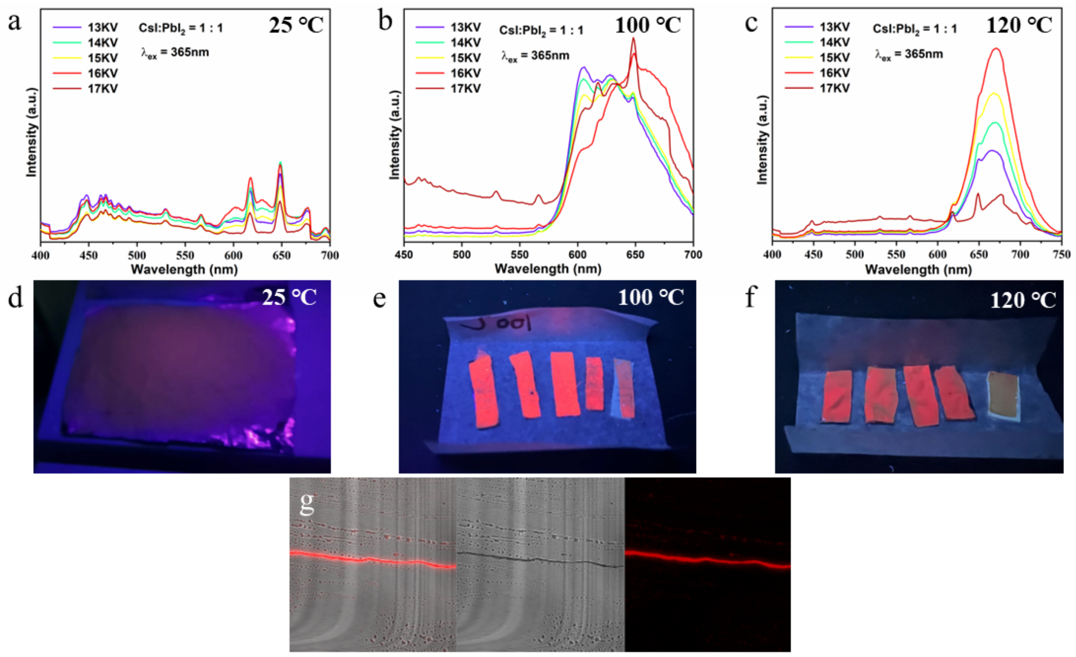

Figure S4. (a-c) PL spectra of $CsPbI_3$/PAN composite nanofibers under different voltages and various thermal treatment temperatures. (d-f) Photos of $CsPbI_3$/PAN composite film under different voltages and various thermal treatment temperatures. (g) LSCM.

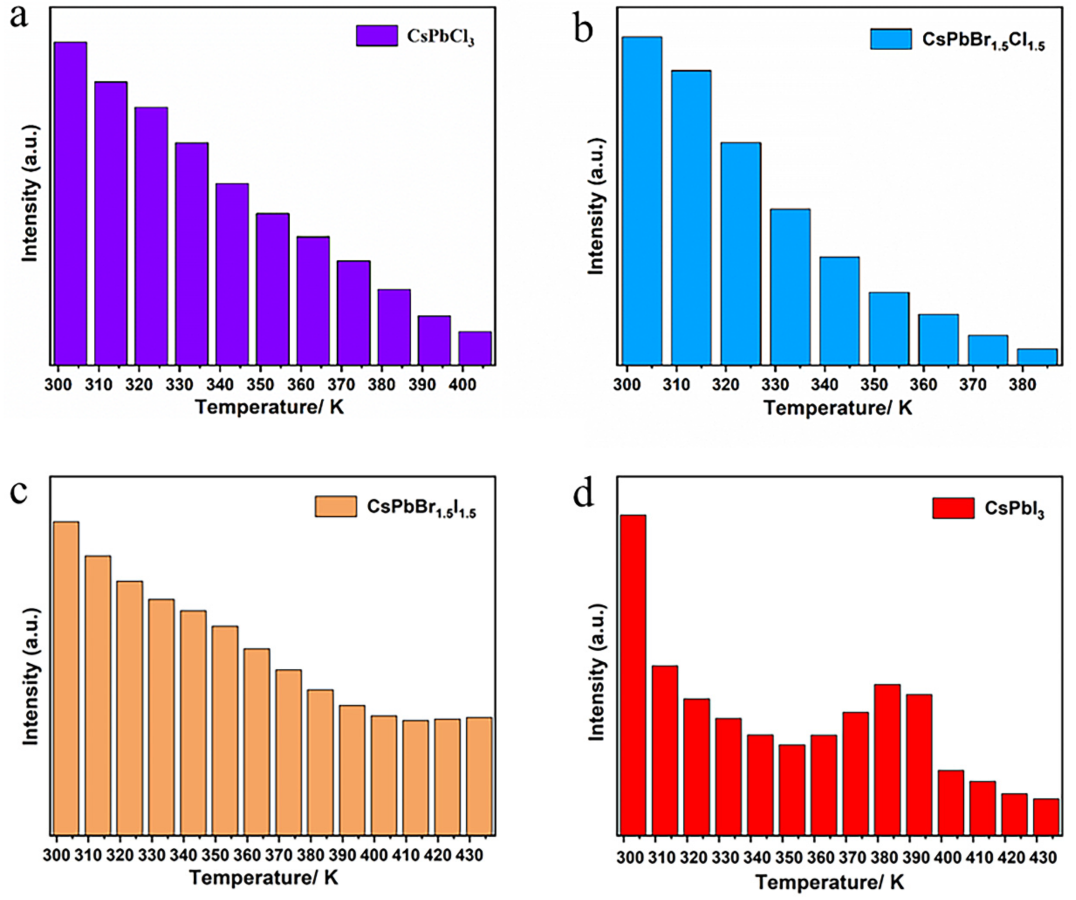

Figure S5. Thermal stability of $CsPbX_3$/PAN composite nanofibers: (a) $CsPbCl_3$/PAN, (b) $CsPbBr_{1.5}Cl_{1.5}$/PAN, (c) $CsPbBr_{1.5}I_{1.5}$/PAN, (d) $CsPbI_3$/PAN.

Table S1. Fluorescence lifetime of multicolor $CsPbX_3$/PAN composite nanofibers.

$CsPbBr_XCl_{3-X}$	Lifetime (ns)	$CsPbBr_XI_{3-X}$	Lifetime (ns)
$CsPbBr_{0.6}Cl_{2.4}$	1.175 ns	$CsPbBr_{2.4}I_{0.6}$	2.428 ns
$CsPbBr_{1.2}Cl_{1.8}$	2.113 ns	$CsPbBr_{1.8}I_{1.2}$	6.516 ns
$CsPbBr_{1.5}Cl_{1.5}$	2.369 ns	$CsPbBr_{1.5}I_{1.5}$	5.450 ns
$CsPbBr_{1.8}Cl_{1.2}$	6.723 ns	$CsPbBr_{1.2}I_{1.8}$	5.999 ns
$CsPbBr_{2.4}Cl_{0.6}$	5.452 ns	$CsPbBr_{0.6}I_{2.4}$	4.684 ns

Table S2. CIE coordinates of multicolor $CsPbX_3$/PAN composite nanofibers.

Samples	CIE Coordinates	Samples	CIE Coordinates
$CsPbCl_3$	(0.205, 0.171)	$CsPbBr_{2.4}I_{0.6}$	(0.207, 0.670)
$CsPbBr_{0.6}Cl_{2.4}$	(0.205, 0.131)	$CsPbBr_{1.8}I_{1.2}$	(0.278, 0.671)
$CsPbBr_{1.2}Cl_{1.8}$	(0.198, 0.104)	$CsPbBr_{1.5}I_{1.5}$	(0.283, 0.610)
$CsPbBr_{1.5}Cl_{1.5}$	(0.176, 0.067)	$CsPbBr_{1.2}I_{1.8}$	(0.368, 0.526)
$CsPbBr_{1.8}Cl_{1.2}$	(0.138, 0.082)	$CsPbBr_{0.6}I_{2.4}$	(0.537, 0.349)
$CsPbBr_{2.4}Cl_{0.6}$	(0.107, 0.526)	$CsPbI_3$	(0.520, 0.326)
$CsPbBr_3$	(0.086, 0.693)		

Table S3. List of water stability tests.

Materials	Duration of the test (Day)	The maintenance of PL intensity (%)	Ref.
$CsPbX_3$-EP	10	85	1
$CsPbBr_3$@APTES@BPSQ	7	82	2
$MAPbBr_3$-3 fibrous mat	2	50	3
$CsPbBr_3$@PVDF-HFP/PS	70	90	4
$CsPbBr_3$/PAN	111	93.5	This work

Reference

1. S. Wei, H. Zhu, J. Zhang, L. Wang, M. An, Y. Wang, X. Zhang, Y. Liu, Luminescent perovskite nanocrystal-epoxy resin composite with high stability against water and air, J. Alloys Compd. 789 (2019) 209-214.

2. C. Ma, M. Zhang, J. Zhang, J. Liao, H. Sun, D. Ji, R. Pang, H. Zhang, J. Liu, S. Liu, Highly Luminescent and Stable Perovskite Quantum Dots Films for Light-Emitting Devices and Information Encryption, Adv. Funct. Mater. 23 (2024) 16717.

3. P.C. Tsai, J.Y. Chen, E. Ercan, C.C. Chueh, S.H. Tung, W.C. Chen, Uniform Luminous Perovskite Nanofibers with Color-Tunability and Improved Stability Prepared by One-Step Core/Shell Electrospinning, Small 14 (2018) 1704379.

4. X. Hu, Y. Xu, J. Wang, J. Ma, L. Wang, W. Jiang, In Situ Fabrication of Superfine Perovskite Composite Nanofibers with Ultrahigh Stability by One-Step Electrospinning Toward White Light-Emitting Diode, Advanced Fiber Materials 5 (2022) 183-197.
